# Characteristics of the Protocols Used in Electrical Pulse Stimulation of Cultured Cells for Mimicking In Vivo Exercise: A Systematic Review, Meta-Analysis, and Meta-Regression

**DOI:** 10.3390/ijms232113446

**Published:** 2022-11-03

**Authors:** Eleni Nintou, Eleni Karligiotou, Maria Vliora, Leonidas G. Ioannou, Andreas D. Flouris

**Affiliations:** FAME Laboratory, Department of Physical Education and Sport Science, University of Thessaly, 382 21 Trikala, Greece

**Keywords:** in vitro, exercise, EPS, cell cultures, muscle contraction

## Abstract

While exercise benefits a wide spectrum of diseases and affects most tissues and organs, many aspects of its underlying mechanistic effects remain unsolved. In vitro exercise, mimicking neuronal signals leading to muscle contraction in vitro, can be a valuable tool to address this issue. Following the Preferred Reporting Items for Systematic Reviews and Meta-Analyses guidelines for this systematic review and meta-analysis, we searched EMBASE and PubMed (from database inception to 4 February 2022) for relevant studies assessing in vitro exercise using electrical pulse stimulation to mimic exercise. Meta-analyses of mean differences and meta-regression analyses were conducted. Of 985 reports identified, 41 were eligible for analysis. We observed variability among existing protocols of in vitro exercise and heterogeneity among protocols of the same type of exercise. Our analyses showed that AMPK, Akt, IL-6, and PGC1a levels and glucose uptake increased in stimulated compared to non-stimulated cells, following the patterns of in vivo exercise, and that these effects correlated with the duration of stimulation. We conclude that in vitro exercise follows motifs of exercise in humans, allowing biological parameters, such as the aforementioned, to be valuable tools in defining the types of in vitro exercise. It might be useful in transferring obtained knowledge to human research.

## 1. Introduction

Voluminous evidence has strongly linked exercise and physical activity levels with improved health, well-being, and quality of life and has shown that they play important roles in the battle against a wide spectrum of multifactorial diseases, such as cancer [1], diabetes [2], osteoporosis [3], cardiometabolic syndrome, and obesity [4,5], in addition to many others. As a result, much research has focused on identifying the molecular and biochemical pathways through which exercise benefits muscle as well as other tissues and organs, such as the adipose tissue, heart [6], brain [7], etc. Although many studies have been conducted to unravel the underlying mechanistic effects of exercise and physical activity, there are still many aspects that remain poorly understood [8]. This limits our understanding of important biological and physiological pathways and inhibits the creation of exercise and physical activity regimes that will have a maximized impact on health, wellbeing, and performance. A more-controlled, “closed” system can contribute to addressing these issues, allowing the study of exercise-induced responses in deeper detail [9]. In this light, it has been suggested that electrical pulse stimulation (EPS) can provide the means to mimic muscle contraction both in vitro and ex vivo [10].

Motor neuron activity comprises both mechanical and electrical signals regulating growth and differentiation processes by affecting both cellular-microenvironment modulation and gene-expression pattern [11]. Such signals can be mimicked by EPS of myotubes in cell culture, which leads to increased contraction and accelerates sarcomere assembly [12], while, at the same time, generating changes in the genetic and metabolic profiles [13]. Hence, EPS represents a valuable tool in exercise research, although the limitation of the probability of non-cell-mediated effects should be taken into consideration [14]. Nevertheless, the substitution of the motor neuron activity with the electrical pulse has been shown to cause changes on myokines and muscle proteins in the cultured skeletal muscles [10] and has been used for tissue engineering [12]. However, the frequency (Hz), pulse duration (ms), applied pulse amplitudes (Vapp), and stimulation duration time of cultured cells in order to achieve exercise-mediating responses are yet to be validated in a systematic way [15].

Published studies have used electrical pulse stimulation to induce acute [16,17,18,19] and chronic [20,21] exercise; aerobic [22], endurance [19,23], and resistance training [24]; and high-intensity [25] and moderate activity [26]. The EPS protocols employed and the validation of the efficacy of the stimulation present a noticeable variability [27]. Moreover, the biological footprint of those models of exercise has been partially evaluated, with the main focus on exercise proteins and myokines, such as Akt (protein kinase B) [16,20,28], AMPK (5’ adenosine monophosphate-activated protein kinase) [23,29,30], and IL-6 (Interleucine 6) [16,25], as well as metabolic indices, mainly glucose metabolism [21,30,31]. Therefore, we did a systematic review and meta-analysis to systematically assess the available evidence on the link between the stated type of exercise and the observed biological profile of exercised cells, as well as to present the available EPS-applied protocols mimicking exercise in vitro.

## 2. Methods

### 2.1. Searching Process

Following the Preferred Reporting Items for Systematic Reviews and Meta-Analyses (PRISMA) guidelines [32] (Table S4), we searched the PubMed and EMBASE databases from their inception to 4 February 2022 for studies that assessed in vitro exercise using EPS as a means to mimic exercise. To increase data availability and method transparency, we uploaded our data to an online repository (https://doi.org/10.6084/m9.figshare.21299523, accessed on 8 October 2022).

The screening of the titles, abstracts, and full texts for eligibility and the selection of studies to be included was performed independently by two investigators (EN and EK). Any conflicts were resolved by a referee investigator (ADF). We included studies where EPS was used to mimic exercise in vitro and the specific type of exercise achieved was defined by the authors. We considered articles written in English published in peer-reviewed journals. No limits were set for methodological design or sample size. We excluded reviews, conference proceedings, editorials, letters, and magazine articles, but we screened the reference lists of such publications of the retrieved articles for relevant papers. Also, we excluded studies without any information on the characteristics of the stimulation protocol (frequency (Hz), pulse duration (ms), and applied pulse amplitudes (Vapp)) [33], on the duration of the stimulation, on the type of the stimulator, and on the cell type that underwent exercise. Moreover, we excluded studies not providing a definition of the type of mimicked exercise and not clearly stating that pulse stimulation was used in order to mimic exercise (therefore, studies where “muscle contraction” was the term used instead of “exercise”). The search algorithm can be found in Supplement 1.1.

### 2.2. Data Extraction

For all eligible studies, we extracted the first author names, year of publication, country of origin, funding acquisition, and data on the pulse parameters, cell type used, and biological indices measured on the cells under stimuli, and we documented the purpose of each study in relationship to the exercise conducted and any relevant secondary outcome (Tables S1–S3). The extracted data are freely available in an online data repository accessed on 8 October 2022 (https://doi.org/10.6084/m9.figshare.21299523). The groups regarding types of exercise studied are based on the definition provided by the authors of each study on the type of exercise achieved, and data was extracted on biological indices.

### 2.3. Meta-Analyses

#### Metanalysis and Meta-Regression

We performed meta-analyses to calculate the differences between control (non-stimulated) and EPS-stimulated cells for the biological indices having enough data for such an analysis. In cases of unreported values, we used WebPlotDigitizer (v4.5, 2021) to extract the information from the given graphs [34]. Meta-regression analysis was used to evaluate the association between duration of stimulation and levels of expression of the examined biological parameters. In cases where the number of replicates was not identified, we assumed that they were conducted in triplicates, and in cases of a range of number of replicates, we used the mean. Since different methods and scales were utilized in the eligible studies, we used standardized mean differences (SMDs) instead of absolute mean differences to standardize our findings to uniform scale [35]. Missing SDs were imputed using the average coefficient of variation from all complete cases [36]. A random effect model was used to account for heterogeneity due to different cell lines, stimulation protocols, and stimulators. All analyses were performed using the “metafor” package in the R language (Rstudio, version 1.3.1093, PBC, Boston, MA, USA). The “**atransf**” **argument** in “metafor” was used for the transformed standardized mean difference as an estimate of the log odds ratio. The level of significance was set at an alpha level of *p* < 0.05.

## 3. Results

### 3.1. General Description of Models

#### 3.1.1. Searching and Selection

**Figure 1:**
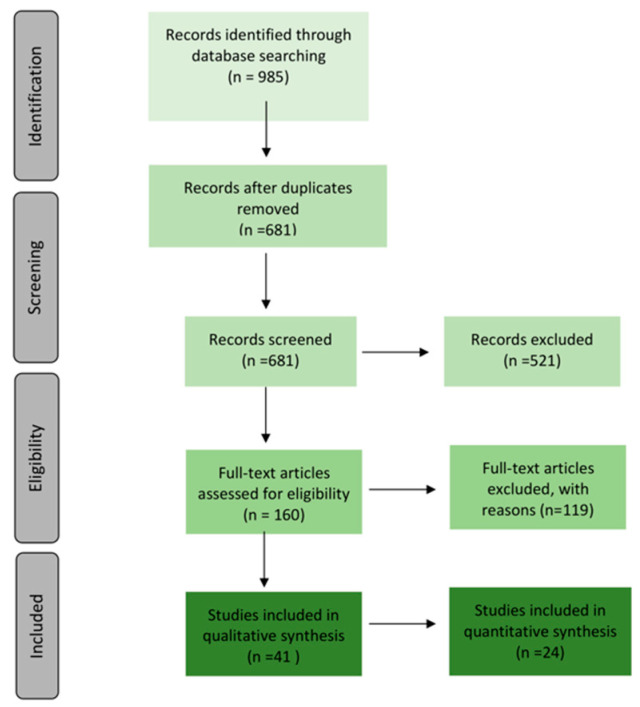
Prisma Flow Chart. The selection process of the studies included in the present systematic review.

A total of 985 records were retrieved through our systematic database search. Of these articles, we removed 308, which were duplicates (Figure 1). An additional 521 records were classified as non-eligible. 161 were assessed for eligibility. Overall, 41 studies met the inclusion criteria. Of these, 24 studies provided information for meta-analysis. The list of included studies and their main outcomes is provided in the Online Supplement (Tables S1–S3).

#### 3.1.2. Cell Types and Pulse-Stimulator Types

Two main groups of cell types were used in the included studies: a. cell lines and b. biopsies from humans and mice (Table S2). More specifically, 30 of the eligible studies used cell lines: 24 studies employed the C2C12 cell line [16,17,26,28,30,31,37,38,39,40,41,42,43,44,45,46,47,48,49,50,51,52,53,54], a mouse myoblast cell line; while one study used the L6 cell line [55], a rat myoblast cell line; one used primary human cells [13]; and one the H-2kb muscle cells (a mouse myoblast cell line) [56]. Of the remaining eligible studies, 12 used human skeletal muscle biopsies [18,19,20,21,23,24,25,29,50,57,58,59,60] from different sites, such as vastus lateralis, satellite cells, and rectus abdominis, obtained from healthy (*n* = 64), lean (*n* = 32), obese (*n* = 20), and diabetic donors (*n* = 4). One study used rat biopsies from the quadriceps [44], while another study used mouse biopsies from 4–8-week-old mice [22] and one rabbit hindlimbs [61]. Also, we identified two main types of electrical pulse stimulators: custom made stimulators (used by 13 studies) and a commercially available stimulator (used by 28 studies). Also, five commercially available generators and electrodes have been reported (Table S2). The eligible studies employed a wide range of electric potential (volts), frequency (Hz), and intensity (amps), while a higher homogeneity was observed in the duration of stimulation (Table S3).

### 3.2. In Vitro Types of Exercise

#### 3.2.1. Acute and Chronic Exercise

A total of 20 studies [16,17,18,19,21,24,25,28,30,31,37,38,41,42,44,45,49,51,53,60] reported that their protocol mimicked acute exercise, and we identified an EPS duration time frame of 15 min to 24 h and one case of repeated stimulation for 3 days, 60 min per day. Almost all (95%) of the protocols mimicking acute exercise included an EPS time period of <100 min. In the case of chronic exercise, the protocols were divided into two major categories. In most studies, chronic exercise was mimicked via a long period of continuous stimulation lasting from 12 to 72 h [20,21,24,25,44,46,54,58], while in some studies chronic exercise was administered as a brief protocol repeated over several consecutive days (3 to 15 days) [38,61].

#### 3.2.2. Aerobic, Resistance, and Endurance Training

McArdle et al. described their exercise as aerobic activity, where the EPS lasted for 15 min (30 V per well), whilst Nieuwoudt et al. (30 V per well) used a protocol consisting of a 16 h stimulation at 11.5 V per mm. In several studies, the type of exercise was defined in a more qualitative way, describing only the type of training mimicked via the applied protocol. In this case, the authors of seven studies [19,23,24,53,55,56,57] reported that their protocol was comparable to resistance exercise. Further analysis of the stimulation parameters showed that six [19,23,24,55,56,57] studies applied the stimulation once (implied as acute) with a range of 15 min to 24 h. Tamura et al. [53], though, used a protocol more similar to that of chronic exercise, applying a 10 min stimulation per day for 3 consecutive days. The protocol used by Breton et al. [24] was the only one where we detected linking both acute (30 min stimulation) and chronic (3 day stimulation) protocols to resistance training in vitro. Furthermore, three studies [39,52,60] identified their EPS model as “endurance training”, either establishing the optimal conditions for EPS to mimic endurance training in vitro (60 min, 11.5 V, 10 Hz) or using an already established protocol (240 min, 20 V, 1 Hz) that was previously proven to mimic endurance exercise in vitro [62].

#### 3.2.3. High-Intensity and Moderate Activity

Regarding the intensity of exercise, eight studies characterized their in vitro exercise models as high-intensity [25,43,47] or mild/moderate [25,26,29,52,56] activity. The remaining studies did not provide relevant information. In one study, a 3D-engineered muscle was employed and an EPS protocol consisting of 30 min, 1 V/mm, and 100 Hz was applied. In the 3D-engineered muscle, the high-intensity in vitro protocol mimicked the muscle fatigue of acute high-intensity exercise in humans.

### 3.3. In Vivo vs. In Vitro

Nine studies [13,17,22,30,39,44,49,50,52] (Table 1) compared their results from exercise mimicking in vitro with their in vivo experiments. A similar pattern of gene expression of MCAD (Medium Chain Acyl CoA Dehydrogenase), Cpt1b ( Carnitine Palmitoytransferase-1b), and GLUT4 ( Glucose transporter type 4) was observed between EPS-treated muscle cells and chronically exercised mice but not in acutely exercised mice [17]. Similarly, phosphorylated AMPKa1/2 was increased in both exercised mice (chronic exercise of 1 h/day for 3 weeks) and stimulated muscle cells (acute and chronic) [44]. A comparison between mice executing treadmill exercise (75% VO_2max_) for 60 min and electrically stimulated myotubes (both considered acute exercise) showed a comparable motif of regulation of Rac1, Axin1, and AMPK [30].

Another approach [52] consisted of comparing the molecular effect of different EPS protocols to that of voluntary wheel running in mice (considered mild endurance exercise), aiming to identify the EPS protocol with the most-similar molecular signature measuring PGC1a (Peroxisome proliferator-activated receptor-gamma coactivator a) levels, AMPK, and p38 phosphorylation. The suggested protocol consisted of 60 min stimulation at 11.5 V and 10 Hz, with a 2 ms pulse stimulus duration.

Pattamapramont and colleagues identified NR4A3 ( Nuclear Receptor Subfamily 4 Group A Member 3) as an exercise-induced gene in acutely exercised healthy men, and then they established an EPS model mimicking the effect of exercise on that particular gene expression. An attempt to map the gene activation pattern of FNDC5A (fibronectin type III domain containing 5a) in EP-stimulated human muscle cells and in human biopsies from participants that either underwent 10-week interval endurance training or 11-week strength training showed no changes in FNDC5 mRNA expression in both exercise models. It should be noted that the EPS protocol was able to enhance PGC1a mRNA expression, which is typical for exercising muscle.

### 3.4. Biological Parameters

Apart from the above-mentioned parameters regarding EPS, the effect of exercise in vitro was evaluated by some authors using exercise-related indicators at biochemical, protein, and translational levels. As previously mentioned, in some studies there was an attempt by authors to correlate biological indices in both in vivo and in vitro experimental setups. These issues are described in the following subsections.

#### 3.4.1. AMPK Signalling

AMPK is phosphorylated in skeletal muscle during exercise due to high binding of AMP, whose concentration (and, therefore, availability) depends on the duration and the intensity of exercise [63]. In this perspective, in 10 of the included studies [16,19,23,28,29,30,31,45,52,53], AMPK and AMP were measured and were found to be increased after the application of EPS compared to controls in all but one [23] study. The protocol was defined as resistance exercise. However, when two types of EPS contraction (both considered by the study authors as resistance exercise), tetanic vs. twitch, were compared, the phosphorylation of the AMPK a-subunit at post-translational modification site Thr172 (regulating AMPK activity) was found to increase significantly in tetanic but not in twitch contraction [53].

#### 3.4.2. Glucose Metabolism

Glucose is the main energy source for exercising skeletal muscle. Glucose availability is determined by the delivery, the transport across the membrane, and the intracellular metabolism; three processes well-orchestrated and tightly connected [64]. Glucose uptake after EPS was measured in eight of the eligible studies: seven studies [21,28,29,31,45,48,53] reported significant increases in glucose uptake, while one study found a decrease after the stimulation [53]. GLUT4H cell surface receptors, which are responsible for glucose transport into the cell, have also been found higher after applying a 60 min acute exercise protocol in C2C12 cells than in the basal condition. In another study, GLUT4-protein expression remained unchanged after a 16 h aerobic-training protocol in C2C12 cells [48]. A 24 h moderate-exercise protocol applied on human biopsies from lean and obese Caucasians increased GLUT4 only in muscle cells from lean individuals [29].

#### 3.4.3. Akt Signalling

Akt signalling pathway is increased by acute bouts of exercise proportionally to the intensity of exercise in human studies, while chronic exercise has minimal effect on Akt activation [65]. In the EPS studies with chronic exercise, Akt levels decreased, while the acute exercise protocols led to an increased phosphorylated Akt [24]. Also, the different timepoints of sample collection seem to play some role, since higher protein levels are detected immediately after the exercise protocol and 180 min later, in contrast to 60 min after the protocol [24].

#### 3.4.4. IL-6 as a Myokine

IL-6 is identified as a myokine secreted by skeletal muscle upon exercise [66] and has been measured in eight of the eligible studies [13,16,19,20,21,23,25,41] at protein and protein-expression levels. Overall, IL-6 secretion increased after the EPS protocol, except for when the muscle cells used were coming from severely obese participants [20]. After a series of measurements over time, Tarum et al. identified a pick at expression levels 4 h after completion of EPS, while, in untreated cells, the IL-6 remained undetected.

### 3.5. Meta-Analyses

#### 3.5.1. Mean Differences in Biological Indices between Stimulated and Non-Stimulated Cells

**Figure 2:**
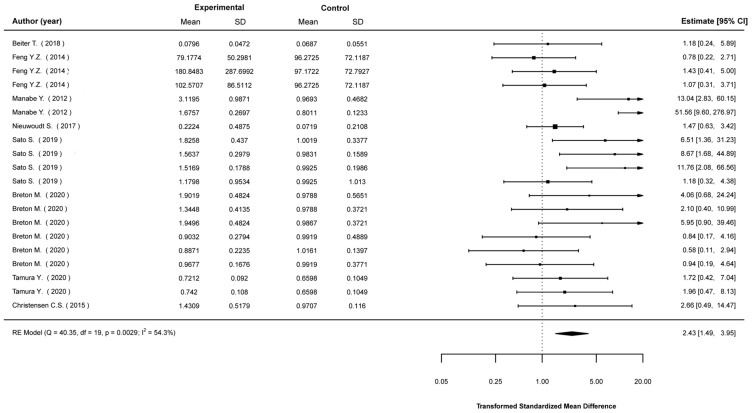
Findings of random-effects meta-analysis on the effects of EPS on Akt compared to non-stimulated cells. Results shown are transformed standardized mean differences and 95% confidence intervals, as an estimate of the log odds ratio. Differences greater than 1.00 favour the EPS cells compared to non-stimulated control cells [16,19,20,24,28,48,53,55].

**Figure 3:**
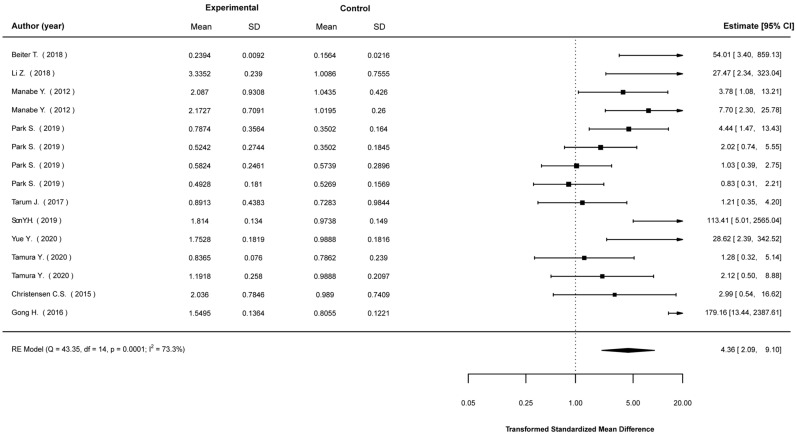
Findings of random-effects meta-analysis on the effects of EPS on AMPK compared to non-stimulated cells. Results shown are transformed standardized mean differences and 95% confidence intervals, as an estimate of the log odds ratio. Differences greater than 1.00 favour the EPS cells compared to non-stimulated control cells [16,19,23,28,29,30,31,45,52,53].

**Figure 4:**
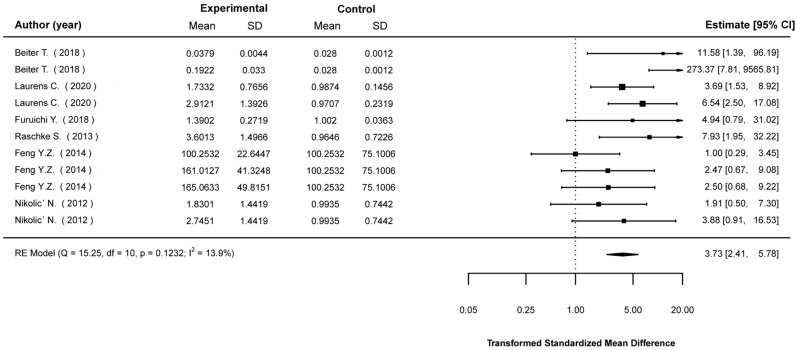
Findings of random-effects meta-analysis on the effects of EPS on IL-6 compared to non-stimulated cells. Results shown are transformed standardized mean differences and 95% confidence intervals, as an estimate of the log odds ratio. Differences greater than 1.00 favour the EPS cells compared to non-stimulated control cells [13,16,20,21,25,41].

**Figure 5:**
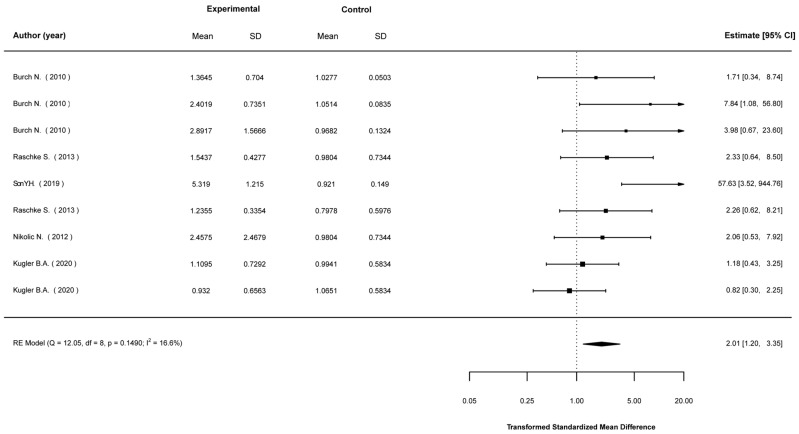
Findings of random-effects meta-analysis on the effects of EPS on PGC1a compared to non-stimulated cells. Results shown are transformed standardized mean differences and 95% confidence intervals, as an estimate of the log odds ratio. Differences greater than 1.00 favour the EPS cells compared to non-stimulated control cells [13,17,21,50,52,58].

**Figure 6:**
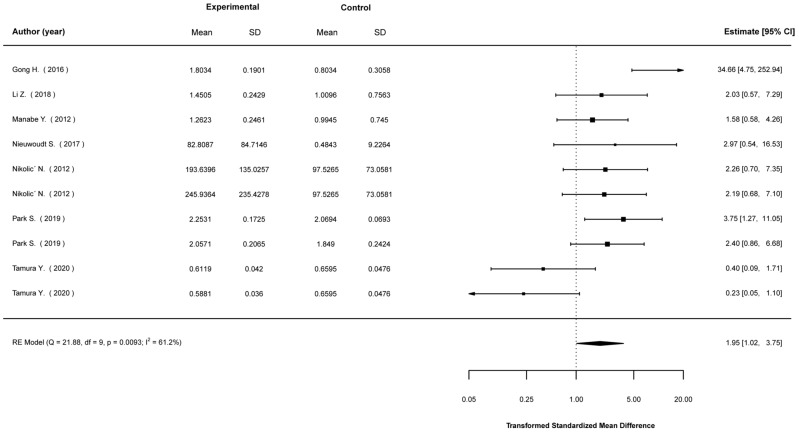
Findings of random-effects meta-analysis on the effects of EPS on glucose uptake compared to non-stimulated cells. Results shown are transformed standardized mean differences and 95% confidence intervals, as an estimate of the log odds ratio. Differences greater than 1.00 favour the EPS cells compared to non-stimulated control cells [21,28,29,31,45,48,53].

**Figure 7:**
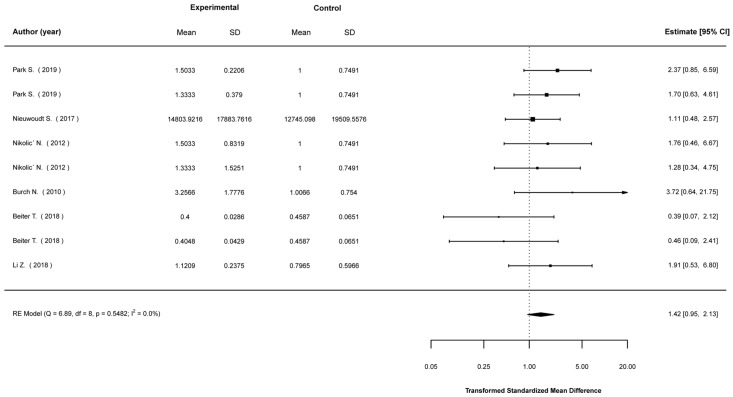
Findings of random-effects meta-analysis on the effects of EPS on GLUT4 compared to non-stimulated cells. Results shown are transformed standardized mean differences and 95% confidence intervals, as an estimate of the log odds ratio. Differences greater than 1.00 favour the EPS cells compared to non-stimulated control cells [16,17,21,29,45,48].

Transformed standardized mean differences between EP-stimulated cells and control (non-stimulated) cells were calculated for the expression levels of Akt, AMPK, IL-6, PGC1-a, and GLUT4, as well as glucose-uptake levels. The analyses showed that EPS cells were much more likely to show higher expression in most of these parameters. Specifically, compared to non-stimulated cells, EPS cells were 2.43 (1.49, 3.95) times (mean (95% CI)) more likely to show higher Akt expression (Figure 2); 4.36 (2.09, 9.10) times more likely to show higher AMPK expression (Figure 3); 3.73 (2.41, 5.78) times more likely to show higher IL-6 expression (Figure 4); 2.01 (1.20, 3.55) times more likely to show higher PGC1a expression (Figure 5); and 1.95 (1.02, 3.75) times more likely to show higher glucose-uptake levels (Figure 6) (all *p* < 0.05). Compared to non-stimulated cells, EPS cells were 1.42 (0.95, 2.13) times more likely to show higher GLUT4 expression, yet this effect did not reach the level of statistical significance (*p* > 0.05; Figure 7).

#### 3.5.2. Meta-Regression for the Effect of EPS Depending on Stimulation Duration

**Figure 8:**
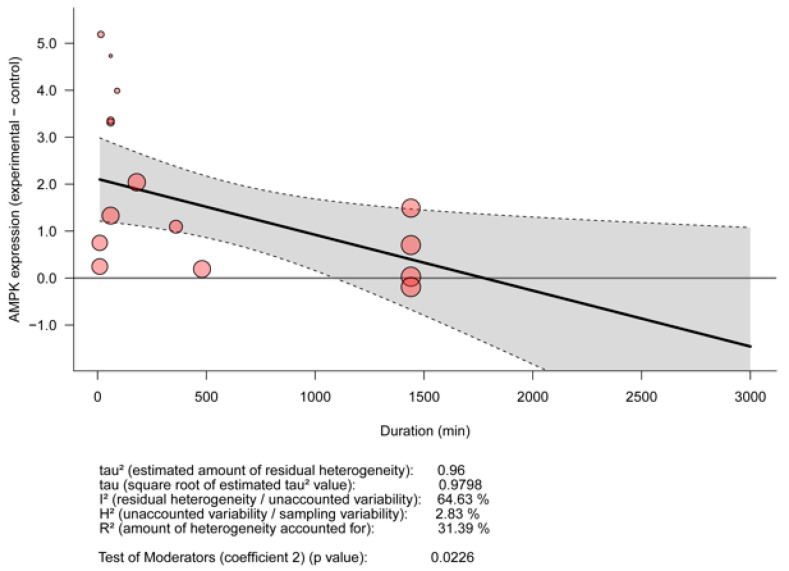

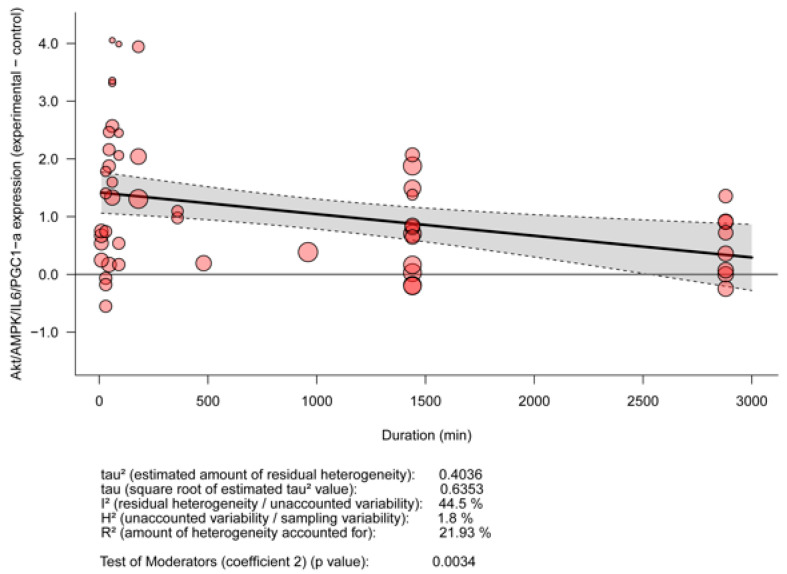
Meta-regression for the effect of EPS depending on stimulation duration in the expression of AMPK (**top**) and combined Akt, AMPK, IL-6, and PGC1a (**bottom**).

The effect of EPS stimulation on AMPK-expression levels was significantly decreased with the duration of stimulation (*p* = 0.023, R^2^ = 0.31; Figure 8). This effect did not reach the level of statistical significance for Akt, IL-6, PGC1a, GLUT4, or glucose uptake (*p* > 0.05; Figures S1–S5). However, when analyzed combined, the overall effect of EPS stimulation on Akt, AMPK, IL-6, and PGC1a also decreased with the duration of stimulation (*p* = 0.034, R^2^ = 0.22; Figure 8).

## 4. Discussion

In the last decades, exercise has been proposed as a prevention and/or therapeutic strategy for many diseases [1,2]. Therefore, much research has focused on identifying the molecular and biochemical pathways through which exercise exerts its benefits. A valuable method to study the underlying mechanisms of exercise effect is in vitro mimicking of exercise via EPS [67].

Differences in terms of exercise intensity, duration, and repetitions lead to different (more or less beneficial) effects [67]. Thus, defining the type of exercise in in vitro experiments is essential both for assessing its overall effect and for highlighting the involved pathways. As shown in this systematic review, there is a vast heterogeneity of applied in vitro protocols reflecting different types of exercise. We recorded types of exercise based on duration (chronic and acute), training (endurance, resistance, and aerobic), and intensity (high, mild, and moderate). We observed marked heterogeneity in the protocols used for the same type of mimicked exercise. Furthermore, we observed marked variability in the in vitro studies that conducted and compared their results with in vivo studies. Specifically, for the acute exercise, there were protocols lasting 60 min, while others lasted 360 min and even 24 h. Similarly, chronic exercise protocols ranged from 12 to 36 h. Added to these differences is the important fact that EPS protocols involve many factors, such as pulse duration (ms), applied pulse amplitudes (Vapp), and stimulation duration time, which exert significant impacts on the final outcome.

One could assume that the protocol parameters define the type of exercise; however, the molecular signature of each protocol might be of equal validity. Our meta-analyses showed that EPS protocols exert significant effects in the expression levels of biological parameters that are known to be affected by exercise in in vivo and human studies. Specifically, we found that EPS leads to significant increases in the expression levels of AMPK, Akt, IL-6, and PGC1a and glucose–uptake levels. The above proteins are involved in major biological processes in skeletal muscle triggered by exercise and muscle contraction [64,68,69,70,71]. More specifically, AMPK is acutely activated in response to exercise [68], and the consequent low-energy status (increased ratio of AMP/ADP: ATP) is involved in metabolic regulation and energy homeostasis by downregulating energy-consuming processes, like fatty acid and cholesterol synthesis, and by upregulating ATP-producing pathways, such as glucose uptake and fatty-acid oxidation [72]. When activated via the Akt/mTORC1 pathway, Akt is key to muscle-mass hypertrophy in the healthy and diseased population [73] and triggered by many extracellular signals, including exercise. IL-6, a pleiotropic myokine, is known to increase in response to exercise exerting both anti- and pro-inflammatory effects [74]. It plays key anti-diabetic roles, enhancing muscular glucose uptake, exerting effects on pancreatic insulin secretion, and promoting fatty-acid oxidation and lipolysis [75]. Upregulation of the p38γ MAPK/PGC-1α pathway and increase of PGC-1α augment mitochondrial biogenesis, fatty-acid oxidation, and insulin sensitivity in healthy and insulin-resistant skeletal muscle, although studies in mice have suggested that PGC1a does not affect insulin sensitivity [76]. Correlating duration of protocol with the mean differences for each of the aforementioned biological indices clearly showed that there was a noteworthy trend for a reduction in the effect of EPS with increasing duration. In particular, the expression of AMPK in stimulated cells significantly decreased with time of stimulation. Likewise, in humans, AMPK has been known to increase in acute exercise and partially in extended chronic exercise [63]. Individually, Akt, IL-6, and PGC1a did not seem to relate with the duration of EPS; although, when analyzed as one group (including AMPK), the effect of stimulation duration became significant. These results may be due to the small number of studies included in our meta-regression but also because the signaling pathways of these molecules are intertwined. For instance, IL-6 has been shown to augment in acute exercise and decrease in plasma of humans both at rest and in response to chronic exercise [77], which is in line with the findings of our meta-regression. Interestingly, glucose uptake and GLUT4 had an opposite trend, increasing with time, meaning that the longer the protocol, the higher the need for glucose uptake and subsequently GLUT4 translocation and expression. Even though AMPK, a regulator of glucose uptake, was found to decrease with time in our meta-regression, glucose uptake changed in the opposite direction, indicating that in vitro models can mimic contraction-induced glucose uptake involving alternative molecular pathways [78].

The present systematic review, meta-analysis, and meta-regression verified previous statements, that in vitro models of exercise have a massive variability in cell types, protocols, equipment, sample collection time, and measurement methods. In this respect, validating in vitro models by comparing the results to those obtained from in vivo studies is of great value [52]. At present, there are a limited number of studies adopting this research design, inhibiting further data analysis and conclusions.

To our knowledge, this is the first time that key biological parameters for exercise are examined in a meta-analysis and meta-regression in relation to their effect in vitro. It is now evident that in vitro exercise follows motifs of exercise in humans, allowing biological parameters, such as AMPK, Akt, IL-6, PGC1a, and glucose uptake to be valuable tools in defining the types of in vitro exercise. Further research is needed to set the base for a consensus that would provide robustness of results and improved translation of the findings into human studies.

## Figures and Tables

**Table 1:** In vitro vs in vivo *studies*. The type of exercise as defined by the study authors and the duration of in vitro exercise. These in vitro types of exercise have been compared directly or indirectly to in vivo models of exercise.

Author, Date	Type of Exercise as Defined by the Study Authors	Duration of In Vitro Exercise	In Vivo Protocol	Organism
Burch, 2010 [17]	Acute, intermittent, continuous	90 min = acute, 90 min/4 days = intermittent, 24 h = continuous	Treadmill, at 75% of average distance of exhaustion trial (4 days training, 1 day exhaustion, 2 days rest), 6 weeks total	Mice
Fernandez-Verdejo, 2017 [39]	Endurance exercise	240 min	Treadmill until exhaustion	Mice
Lee, 2020 [44]	Acute and chronic exercise	Acute = 1, 3, 6 h chronic = 12, 24, or 36 h	Treadmill 60 min, 5 d/week, 10 m/min	Mice
McArdle, 2001 [22]	Aerobic activity	15 min		
Pattamaprapanont, 2016 [49]	Acute exercise	30 min	Cycle ergometer at 80% VO_2max_, 15 min	Healthy males
Raschke, 2013 [13]	Regular exercise	4 to 24 h	Cycle ergometer at 70% VO_2max_, 60 min	Healthy males
Raschke, 2013 [50]	Training model/in humans endurance training	24 h	Treadmill, at 90% of peak heart rate, 3 d/week for 10 weeks	Healthy males
Son, 2019 [52]	Mild endurance exercise	60 min	Volunteer wheel running daily for 4 weeks	Mice
Yue, 2020 [30]	Acute exercise	60 min	Treadmill, at 75% VO_2max_, 60 min	Mice

## Data Availability

Data supporting reported results can be found at https://doi.org/10.6084/m9.figshare.21299523, accessed on 8 October 2022.

## Supplementary Materials

The following supporting information can be downloaded at: https://www.mdpi.com/article/10.3390/ijms232113446/s1. References [13,16,17,18,19,20,21,22,23,24,25,26,28,29,30,31,37,38,39,40,41,42,43,45,46,47,48,49,50,51,52,53,54,55,56,57,58,59,60,61,79] are cited in the supplementary materials.
