# Supplementary material for: Characteristics of the Protocols Used in Electrical Pulse Stimulation of Cultured Cells for Mimicking In Vivo Exercise: A Systematic Review, Meta-Analysis, and Meta-Regression
Source: Int J Mol Sci. 2022 Nov 3;23(21):13446. doi: 10.3390/ijms232113446 (PMC9657802; doi:10.3390/ijms232113446)
Supplement: Supplementary file 1 [file ijms-23-13446-s001.zip › ijms-1987957-supplementary.pdf]

Characteristics of the protocols used in electrical pulse stimulation of cultured cells for mimicking in vivo exercise: A systematic review, meta-analysis and meta-regression

Eleni Nintou, Eleni Karligiotou, Maria Vliora, Leonidas G. Ioannou and Andreas D. Flouris

FAME Laboratory, Department of Physical Education and Sport Science, University of Thessaly, Trikala, Greece

Corresponding author: andreasflouris@gmail.com (A.D.F.)

S1.1 Materials and Methods

S1.1.1. Search Strategy

The following algorithm has been used both in EMBASE and PubMed and was accordingly modified, when needed.
The search term combination was the following for both databases:

((((((((((((((pulse*[Title/Abstract])) OR (electric*[Title/Abstract])) OR (stimul*[Title/Abstract])) OR (contract*[Title/Abstract])) OR (frequency[Title/Abstract])) OR (electrode*[Title/Abstract])) OR (field[Title/Abstract])) OR (train[Title/Abstract])) OR (bipolar[Title/Abstract])) OR (pacemaker[Title/Abstract])) OR (c-pace[Title/Abstract]))) AND (((((((((((cell line*[Title/Abstract])) OR (cell culture*[Title/Abstract])) OR (cell*[Title/Abstract])) OR (musc*[Title/Abstract])) OR (myotube*[Title/Abstract])) OR (myoblast*[Title/Abstract])) OR (muscle cell*[Title/Abstract])) OR (myofiber*[Title/Abstract])) OR (skeletal[Title/Abstract])) OR (myofibril*[Title/Abstract])) OR (contractile activity[Title/Abstract]))) AND ((((physical activity[Title/Abstract])) OR (exercise[Title/Abstract])) OR (training[Title/Abstract]))) AND (((in vitro[Title/Abstract])) NOT (in vivo[Title/Abstract]))

S1.1.2. Data extraction

Main outcomes and characteristics of the included studies are presented in the following tables.

Table S1. Main outcomes of the included studies

Author, Year	Results- Outcomes
Barlow, 2019 [1]	Molecules released as a consequence of acute skeletal muscle contraction, may improve insulin secretion by cells in obese prediabetic and T2D patients who have controlled hyperglycaemia
Beiter T., 2018 [2]	The cell culture system does not allow to directly monitor long-term adaptation processes following endurance exercise training, but is quite suitable to analyze acute contraction
Blas A. Guigni, 2019 [3]	Results suggest that the beneficial effect of electrical field stimulation derives from the activation of mechanotransductive pathways that downregulate proteolysis and preserve mitochondrial content protect against the atrophic effects of chemotherapeutics
Broholm C., 2011 [4]	Leukemia Inhibitory Factor (LIF) mRNA is induced in human skeletal muscle following resistance exercise and LIF protein is secreted from electrically stimulated cultured myotubes
Burch N., 2010 [5]	EPS of muscle cells in culture triggers an increase in fatty acid b-oxidation resembling the adaptations of this pathway in chronic endurance exercise. Future combining EPS with mechanical stretch or temporary hypoxia might further help to approximate the environment a fiber a trained muscle's fibre is exposed to
Christensen C.S., 2015 [6]	Due to the lack of blood flow, innervation and skeletal connection the EPS HSkM model cannot precisely mimic in vivo exercise. However, the protocol applied elicited molecular adaptations that more likely resemble resistance exercise
Laurens C., 2020 [7]	GDF15 is a potentially novel exerkine produced by skeletal muscle contraction and able to target human adipose tissue to promote lipolysis
Connor M.K., 2001 [8]	Studies revealed elevated DNA binding in response to contractile activity. This was paralleled by increases in Sp1 protein levels. Variations in the rate of mitochondrial ATP synthesis are important in determining cytochrome c gene expression in muscle cells and this is mediated, in part, by Sp1-induced increases in cytochrome c transcription
Feng YZ., 2014 [9]	EPS enhanced oxidative capacity of glucose in myotubes from all subjects, in contrast to oleic acid that affected only in lean subjects. Human myotubes display the same phenotype as intact muscle in vivo
Fernández-Verdejo R., 2017 [10]	ATF3 is induced by EPS and regulates chemokine mRNA expression in C2C12 myotubes. Part of the ATF3 up-regulation in contracting skeletal muscle occurs in myofibers
Fujita H., 2010 [11]	Contractile activity not only enhances myotube maturation in vitro, but additionally induces changes in the sense of a fast-to-slow transition. Successfully managed to have artificially exercised C2C12 myotubes

Furuichi Y., 2018 [12]	The secretion of IL-6 increased, following muscle contraction, in the absence of cellular damage, suggesting the presence of a secretory machinery in skeletal muscle cells
Gong H., 2016 [13]	EPS stimulation significantly increased intracellular ATP levels
Horie M., 2015 [14]	EPS induced excessive ROS production in contracting C2C12 myotubes and metabolism of ROS resulting from Nrf2 activation protected the myotubes from EPS-induced apoptosis
Kubis HP., 2002 [15]	A ON period of 5 min in a 45 min stimulation cycle is sufficient to induce MHCI e pression and reduce MHCIId expression (mRNA and protein levels). Shorter ON periods of 1.5 min in a 45 min cycle failed to induce a fast-to-slow transition
Lambertucci RH., 2012 [16]	Moderate electrical stimulation increases ROS and NO production by primary rat skeletal muscle cells
Lee J.O..,2020 [17]	Acute and chronic EPS increased the secretion and expression of metrnl into conditioning media and cell lysates and the phosphorylation of AMPK2a in C2C12 myotubes . Metrnl improves glucose metabolism via AMPK-2 and is a promising therapeutic candidate for glucose-related diseases such as type 2 diabetes
Li Z., 2018 [18]	Acute myotube contraction activates signaling of LKB1, AMPK and CaMKII to increase surface GLUT4 levels
Manabe Y., 2012 [19]	C2C12 model is suitable for defining the physiological role of intracellular signaling evoked by muscle contraction
Martin N.R.W., 2017 [20]	Leucine supplementation may augment skeletal muscle functional capacity, validates the use of engineered skeletal muscle for highly-controlled investigations into nutritional regulation of muscle physiology.
McArdle F., 2001 [21]	The short period of contractile activity induced a significant rise in the superoxide level detected in muscle interstitial fluid by microdialysis techniques, but did not induce any significant damage to skeletal muscle fibers
Nieuwoudt S., 2017 [22]	EPS protected against palmitate-induced reductions in PI3K activity, despite the reduction in enzyme activity. Also the respond to contraction stimuli happened in a predictable manner. Contraction alone may protect muscle from lipid-induced insulin resistance
Nikolic´ N., 2012 [23]	EPS did not induce toxic effects to cultured human skeletal muscle cells and in chronic continuous, low-frequency EPS, improved lipid oxidation and glucose metabolism and a possible fiber-type switch was detected. *In vitro* EPS (Acute, high-frequent as well as chronic, low-frequent) of human myotubes may be used to study effects of exercise
Løvsletten N., 2019 [24]	Challenging the cells with EPS lead to different responses in myotubes from non-diabetic vs. diabetic subjects

Park S.,2019 [25]	Electrical pulse stimulation of primary myotubes from lean and severely obese subjects induced improvements in insulin action, but to a lesser extent in those with severe obesity
Pattamaprapanont P.,2016 [26]	This EPS model can mimic some, but not all the features of muscle contraction in vivo
Pattwell DM., 2004 [27]	Skeletal muscle cells release multiple ROS during contractile activity and the pattern of release differs depending on the nature of the ROS species and the frequency of stimulation. The reduction of cytochrome c in the supernatant of muscle cells is not related to the number of contractions undertaken or the frequency of stimulation
Raschke S.,2013 [28]	DDP4 and PEDF were identified to be also secreted by skeletal muscle cells. The release of 45 myokines is regulated by contraction and among these factors, 18 are described as myokines for the first time.
Raschke S. 2013 [29]	This EPS model significantly enhanced PGC1a mRNA expression
Sato S., 2019 [30]	The newly established in vitro muscle contraction model is suitable for analyzing the activation of mTORC1 signaling pathway in cultured L6.C11 myotubes
Scheler M., 2013 [31]	In vitro exercise model can be used to identify exercise-regulated myokines and can be applied to primary human myotubes to study molecular mechanisms of the individual outcome of exercise intervention
Tarum J., 2017 [32]	The study demonstrates that EPS is an *in vitro* exercise model promoting the hypertrophy of human muscle cells, recapitulating a major physiological end-point to resistance exercise in human skeletal muscle
Thelen M.H., 1997 [33]	The opposing stimuli of T_3 and (chronic) contractile activity determine the expression of SERCA1, a typical fast isoform, in skeletal muscle
Son Y.H., 2019 [34]	High similarity and correlation observed for most parameters between EPS and VWR. Various EPS conditions induce muscle hypertrophy and mitochondrial biogenesis, which are phenotypes displayed in resistance and endurance exercise
Yue Y., 2019 [35]	Acute EPS-induced myotube contraction or treadmill exercise regulated Axin1 protein expression in a manner dependent on AMPK activation, while stimulation of Rac1 is AMPK-dependent in both contracted myotubes and exercised skeletal muscle
Valero-Breton M., 2020 [36]	The parameters of stimulation developed could be useful for future studies intending to investigate the molecular responses of acute and chronic resistance exercise in an in vitro model in the quest to develop exercise-mimetics.
Chaves A.B., 2021 [37]	Acute aerobic exercise was able to significantly reduce T NIP and REDD1 protein expression, which may be mediated by a PKA- or cAMP-related mechanism, as indicated by the in vitro experiments

Kugler B.A., 2021 [38]	24 hours of EPS resulted in an improved mitochondrial network structure towards fusion in myotubes derived from lean humans and humans with severe obesity, which was associated with improved skeletal muscle insulin signaling
Nakamura T., 2021 [39]	The findings suggested that exposure of 3D-engineered muscle to acute EPS mimicked muscle fatigue during acute high-intensity exercise in humans
Small L., 2020 [40]	Results of the study suggest that a proportion of the ability of exercise to entrain the skeletal muscle clock driven directly by muscle contraction. Contraction Interventions may be used to mimic some time of day specific effects of exercise on metabolism and muscle performance
Tamura Y., 2020 [41]	From a qualitative perspective, tetanus and twitch were shown to promote metabolic adaptation in the same direction

Table S2. Main extracted data. A general description of the included studies. In the table are shown the cell types, pulse stimulator types, duration of exercise and the type of in vitro exercise.

#	First author, date			C2C12/L6/H2k	Human skeletal muscle biopsies	Primary rat/mouse/rabbit cells	Primary human cells	Custom made stimulator	Commercially available	Duration of stimulation	Acute	Chronic	Aerobic	Endurance	Resistance	High intensity	Moderate
1	Barlow J., 2019 [1]			√					√	64 min	√						
2	Beiter T., 2018 [2]			√					√	90 min	√						
3	Blas A. Guigni, 2019 [3]			√					√	60 min	√						
4	Broholm C., 2011 [4]				√				√	180 min					√		
5	Burch N., 2010 [5]			√					√	90 min, 90 min daily/ 4 consecutive days,24 hrs	√	√					
6	Christensen C.S., 2015 [6]				√				√	360 min	√				√		
7	Laurens C., 2020 [7]				√				√	180min and 24h	√	√				√	√
8	Connor M.K., 2001 [8]			√				√		5, 15, 30, 60, or 240 min in one day, 180 min/day	√	√					
9	Feng Y.Z., 2014 [9]				√			√		48 hrs		√					
10	Fernández-Verdejo R., 2017 [10]			√					√	240min				√			
11	Fujita H., 2010 [11]			√					√	7-11 days							
12	Furuichi Y., 2018 [12]			√					√	60min	√						
13	Gong H., 2016 [13]			√				√		1-15 min	√						
14	Horie M., 2015 [14]			√					√	60min, 180min, 360min						√	

15	Kubis H.P., 2002 [15]					√		√		cycles of 45 min		√					
16	Lambertucci R.H., 2012 [16]					√		√		60min							√
17	Lee J.O..,2020 [17]			√					√	60min, 180 min, 360 min, 12hrs, 24hrs, or 36 hrs							
18	Li Z., 2018 [18]			√					√	60 min	√						
19	Manabe Y., 2012 [19]			√					√	60 min, 180 min	√						
20	Martin N.R.W., 2017 [20]			√				√		24 hrs		√					
21	McArdle A., 2001[21]					√		√		15 min			√				
22	Nieuwoudt S., 2017 [22]			√					√	16 hrs			√				
23	Nikolic´ N., 2012 [23]				√			√		5–60 min, 12 hrs, 24hrs	√	√					
24	Løvsletten N., 2019 [24]				√				√	24 hrs							
25	Park S.,2019 [25]				√				√	24 hrs							√
26	Pattamaprapanont P.,2016 [26]			√					√	30 min	√						
27	Pattwell D.M., 2004 [27]			√				√		15 min	√					√	
28	Raschke S.,2013 [28]						√		√	4 to 24 hrs							√
29	Raschke S. 2013 [29]			√			√		√	24 hrs							
30	Sato S., 2019 [30]			√					√	45min				√			
31	Scheler M., 2013 [31]				√				√	2hr, 4hrs, 8hrs, 24 hrs	√						
32	Tarum J., 2017 [32]				√				√	8 hrs				√			
33	Thelen M.H., 1997 [33]			√				√		48 hrs		√					
34	Son Y.H., 2019 [34]			√					√	60 min				√			√
35	Yue Y., 2019 [35]			√					√	60 min	√						
36	Breton M., 2020 [36]				√				√	30 min, 3 days	√	√			√		
37	Chaves A.B., 2022[37]				√				√	24 hrs	√						
38	Kugler B.A., 2020 [38]				√				√	24 hrs		√					
39	Nakamura T., 2021 [39]			√					√	30 min	√					√	
40	Small L., 2020 [40]			√					√	30 min	√						

41	Tamura Y., 2020 [41]			√					√	10 min					√		

Table S3. Pulse stimulation characteristics of the included studies

	Pulse duration	Pulse amplitude	Frequency
Barlow, 2019 [1]	2 ms pulses	40 V	1 Hz
Beiter T., 2018 [2]	2 ms pulses	14 V	1 Hz
Blas A. Guigni, 2019 [3]	12 ms pulses	20 V	1 Hz
Broholm C., 2011 [4]	1 ms pulses	40 V	1 Hz
Burch N., 2010 [5]	1 ms pulses	14 V	50 Hz
Christensen C.S., 2015 [6]	2 ms pulses	40 V	1Hz
Laurens C., 2020 [7]	Acute=24-ms pulses / chronic = 2-ms pulses	10 V	Acute=0.5 Hz/ Chronic= 0.1 Hz
Connor M.K., 2001 [8]		65 V	5 Hz
Feng YZ., 2014 [9]	2 ms pulses	30 V	1 Hz
Fernández-Verdejo R., 2017 [10]	2 ms pulses	20 V	1 Hz
Fujita H., 2010 [11]	twitch contraction: 10 ms	1 V/mm	50 Hz
Furuichi Y., 2018 [12]	3ms, 30ms, 50ms pulse duration	various voltages	1-Hz
Gong H., 2016 [13]	11 ms pulses	30 V	1, 4, 10, 30 Hz
Horie M., 2015 [14]	2 ms pulses	14, 20, and 40 V	1 Hz
Kubis HP., 2002 [15]	2.5 ms pulses		II, III & V=1, IV=5 or I=10 Hz
Lambertucci RH., 2012 [16]		5 V	50 Hz
Lee J.O.,2020 [17]	1 ms pulses	25 V	1 Hz
Li Z., 2018 [18]	24 ms pulses	20 V	1 Hz
Manabe Y., [19]	3 ms pulses	50 V	1 Hz
Martin N.R.W., 2017 [20]		1 V/mm	10 Hz
McArdle F., 2001 [21]	2 ms pulses	30 V/well	1Hz
Nieuwoudt S., 2017 [22]		1.5 V/mm	1Hz, 0.5 Hz

Nikolic´ N., 2012 [23]	Acute=200 ms given every 5th second/ chronic=2 ms	50 V	Acute= 100 Hz/chronic=1 Hz
Løvsletten N., 2019 [24]	2ms pulses	10 V	0,1 Hz
Park S.,2019 [25]	2 ms pulses	11.5 V	1 Hz
Pattamaprapanont P.,2016 [26]	2 ms pulses	30 V	1 Hz
Pattwell DM., 2004 [27]	2 ms in duration for 0.5 of a second every 5 s	30V/well	1 Hz or 50 Hz
Raschke S.,2013 [28]	2 ms pulses	11.5 V	1 Hz
Raschke S. 2013 [29]	2 ms pulses	11.5 V	1 Hz
Sato S., 2019 [30]	2 ms pulses	50 V	100 Hz
Scheler M., 2013 [31]	2ms pulses	4 V and 14 V	5 Hz
Tarum J., 2017 [32]	2 ms pulses	12 V	1 Hz
Thelen M.H., 1997 [33]	6 ms pulses	3 V/cm2	2Hz
Son Y.H., 2019 [34]	1 ms pulse of 2 ms duration	11.5 V	10 Hz
Yue Y., 2019 [35]	24 ms at 976-ms intervals	20 V	1 Hz
Valero-Breton M., 2020 [36]	0.4 ms with 4 s rest between each contraction	15 V	100 Hz
Chaves A.B., 2021 [37]	2 ms	11.5 V	1 Hz
Kugler B.A., 2021 [38]	2 ms pulses	11.5 V	1 Hz
Nakamura T., 2021 [39]	2 ms pulses	1 V/mm	100 Hz
Small L., 2020 [40]	2 ms pulses	30 V	1 Hz
Tamura Y., 2020 [41]	2 ms,<br>twitch and tetanus	13 V,<br>twitch and tetanus	twitch:2 Hz (continuous) and tetanus: 66 Hz (5s ON, 5 s OFF)

S2.1. Metanalytic findings

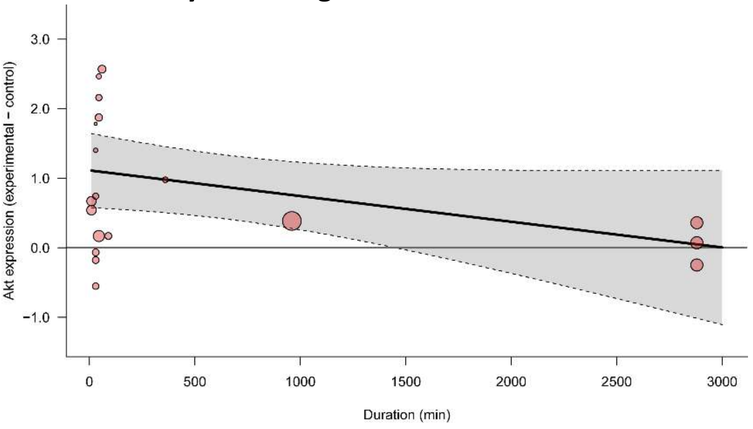

tau² (estimated amount of residual heterogeneity): 0.5207
tau (square root of estimated tau² value): 0.7216
I² (residual heterogeneity / unaccounted variability): 48.5 %
H² (unaccounted variability / sampling variability): 1.94 %
R² (amount of heterogeneity accounted for): 19.82 %

Test of Moderators (coefficient 2) (p value): 0.0906

Figure S1. Correlation of the duration of stimulation with the difference in expression levels of Akt.

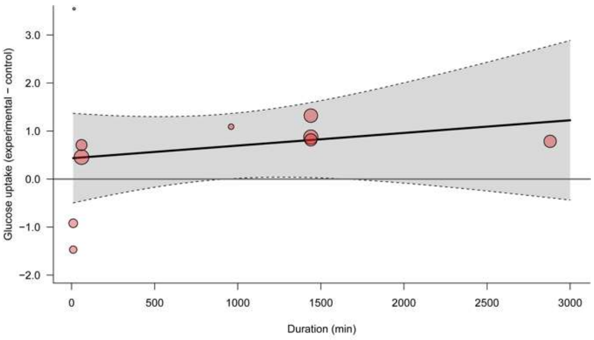

tau² (estimated amount of residual heterogeneity): 0.7363
tau (square root of estimated tau² value): 0.8581
I² (residual heterogeneity / unaccounted variability): 63.44 %
H² (unaccounted variability / sampling variability): 2.74 %
R² (amount of heterogeneity accounted for): 0 %

Test of Moderators (coefficient 2) (p value): 0.4742

Figure S2. Correlation of the duration of stimulation with the difference in expression levels of Glucose Uptake.

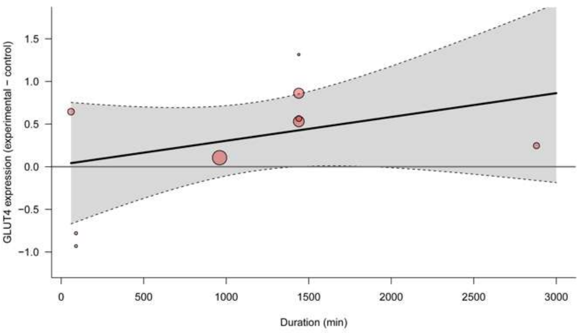

tau² (estimated amount of residual heterogeneity): 0
tau (square root of estimated tau² value): 0
I² (residual heterogeneity / unaccounted variability): 0 %
H² (unaccounted variability / sampling variability): 1 %
R² (amount of heterogeneity accounted for): 100 %

Test of Moderators (coefficient 2) (p value): 0.3016

Figure S3. Correlation of the duration of stimulation with the difference in expression levels of GLUT4.

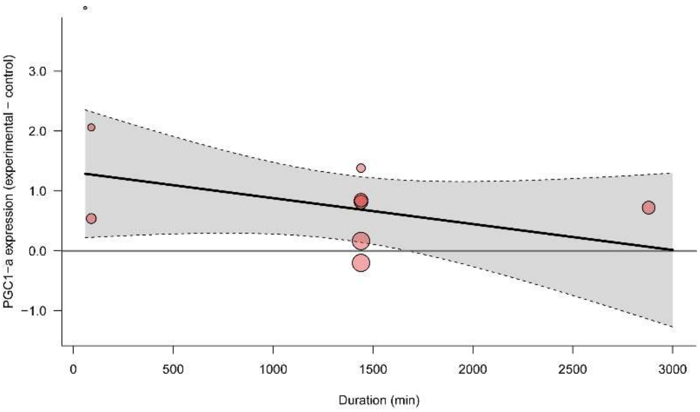

tau² (estimated amount of residual heterogeneity): 0.1672
tau (square root of estimated tau² value): 0.4089
I² (residual heterogeneity / unaccounted variability): 25.19 %
H² (unaccounted variability / sampling variability): 1.34 %
R² (amount of heterogeneity accounted for): 0 %

Test of Moderators (coefficient 2) (p value): 0.2308

Figure S4. Correlation of the duration of stimulation with the difference in expression levels of PGC1a.

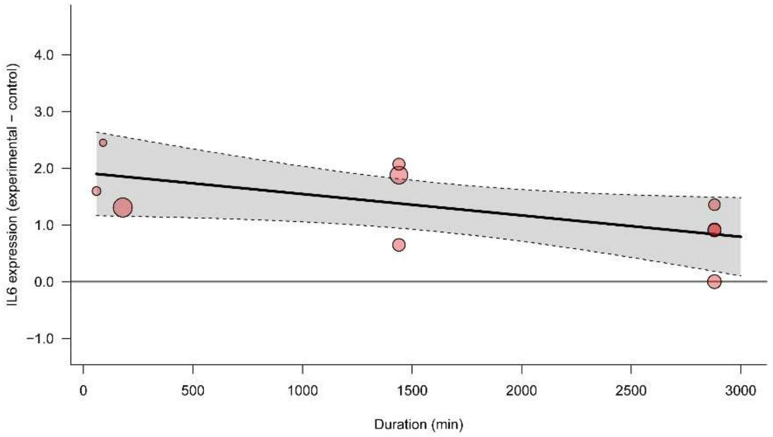

tau² (estimated amount of residual heterogeneity): 0.061
tau (square root of estimated tau² value): 0.2469
I² (residual heterogeneity / unaccounted variability): 11.14 %
H² (unaccounted variability / sampling variability): 1.13 %
R² (amount of heterogeneity accounted for): 19.37 %

Test of Moderators (coefficient 2) (p value): 0.0562

Figure S5. Correlation of the duration of stimulation with the difference in expression levels of IL-6.

S3.1. PRISMA checklist

Table S4. Prisma checklist

Section and Topic	Item #	Checklist item	Location where item is reported
**TITLE**			
Title	1	Identify the report as a systematic review.	1
**ABSTRACT**			
Abstract	2	See the PRISMA 2020 for Abstracts checklist.	1
**INTRODUCTION**			
Rationale	3	Describe the rationale for the review in the context of existing knowledge.	1,2
Objectives	4	Provide an explicit statement of the objective(s) or question(s) the review addresses.	2
**METHODS**			
Eligibility criteria	5	Specify the inclusion and exclusion criteria for the review and how studies were grouped for the syntheses.	11
Information sources	6	Specify all databases, registers, websites, organisations, reference lists and other sources searched or consulted to identify studies. Specify the date when each source was last searched or consulted.	11
Search strategy	7	Present the full search strategies for all databases, registers and websites, including any filters and limits used.	11
Selection process	8	Specify the methods used to decide whether a study met the inclusion criteria of the review, including how many reviewers screened each record and each report retrieved, whether they worked independently, and if applicable, details of automation tools used in the process.	11
Data collection process	9	Specify the methods used to collect data from reports, including how many reviewers collected data from each report, whether they worked independently, any processes for obtaining or confirming data from study investigators, and if applicable, details of automation tools used in the process.	12
Data items	10a	List and define all outcomes for which data were sought. Specify whether all results that were compatible with each outcome domain in each study were sought (e.g. for all measures, time points, analyses), and if not, the methods used to decide which results to collect.	S1-S7
	10b	List and define all other variables for which data were sought (e.g. participant and intervention characteristics, funding sources). Describe any assumptions made about any missing or unclear information.	11,12
Study risk of bias assessment	11	Specify the methods used to assess risk of bias in the included studies, including details of the tool(s) used, how many reviewers assessed each study and whether they worked independently, and if applicable, details of automation tools used in the process.	11,12
Effect measures	12	Specify for each outcome the effect measure(s) (e.g. risk ratio, mean difference) used in the synthesis or presentation of results.	11, 12
Synthesis methods	13a	Describe the processes used to decide which studies were eligible for each synthesis (e.g. tabulating the study intervention characteristics and comparing against the planned groups for each synthesis (item #5)).	11,12
	13b	Describe any methods required to prepare the data for presentation or synthesis, such as handling of missing summary statistics, or data conversions.	11,12

Section and Topic	Item #	Checklist item	Location where item is reported
	13c	Describe any methods used to tabulate or visually display results of individual studies and syntheses.	11, 12
	13d	Describe any methods used to synthesize results and provide a rationale for the choice(s). If meta-analysis was performed, describe the model(s), method(s) to identify the presence and extent of statistical heterogeneity, and software package(s) used.	11,12
	13e	Describe any methods used to explore possible causes of heterogeneity among study results (e.g. subgroup analysis, meta-regression).	11,12
	13f	Describe any sensitivity analyses conducted to assess robustness of the synthesized results.	11,12
Reporting bias assessment	14	Describe any methods used to assess risk of bias due to missing results in a synthesis (arising from reporting biases).	-
Certainty assessment	15	Describe any methods used to assess certainty (or confidence) in the body of evidence for an outcome.	-
**RESULTS**			
Study selection	16a	Describe the results of the search and selection process, from the number of records identified in the search to the number of studies included in the review, ideally using a flow diagram.	11,12
	16b	Cite studies that might appear to meet the inclusion criteria, but which were excluded, and explain why they were excluded.	
Study characteristics	17	Cite each included study and present its characteristics.	S1-S7
Risk of bias in studies	18	Present assessments of risk of bias for each included study.	-
Results of individual studies	19	For all outcomes, present, for each study: (a) summary statistics for each group (where appropriate) and (b) an effect estimate and its precision (e.g. confidence/credible interval), ideally using structured tables or plots.	2-6
Results of syntheses	20a	For each synthesis, briefly summarise the characteristics and risk of bias among contributing studies.	-
	20b	Present results of all statistical syntheses conducted. If meta-analysis was done, present for each the summary estimate and its precision (e.g. confidence/credible interval) and measures of statistical heterogeneity. If comparing groups, describe the direction of the effect.	3-6, 7-10
	20c	Present results of all investigations of possible causes of heterogeneity among study results.	10
	20d	Present results of all sensitivity analyses conducted to assess the robustness of the synthesized results.	6-10
Reporting biases	21	Present assessments of risk of bias due to missing results (arising from reporting biases) for each synthesis assessed.	-
Certainty of evidence	22	Present assessments of certainty (or confidence) in the body of evidence for each outcome assessed.	6-10
**DISCUSSION**			
Discussion	23a	Provide a general interpretation of the results in the context of other evidence.	10
	23b	Discuss any limitations of the evidence included in the review.	10
	23c	Discuss any limitations of the review processes used.	10
	23d	Discuss implications of the results for practice, policy, and future	10

Section and Topic	Item #	Checklist item	Location where item is reported
		research.	
**OTHER INFORMATION**			
Registration and protocol	24a	Provide registration information for the review, including register name and registration number, or state that the review was not registered.	12
	24b	Indicate where the review protocol can be accessed, or state that a protocol was not prepared.	S1, 12
	24c	Describe and explain any amendments to information provided at registration or in the protocol.	12
Support	25	Describe sources of financial or non-financial support for the review, and the role of the funders or sponsors in the review.	12
Competing interests	26	Declare any competing interests of review authors.	12
Availability of data, code and other materials	27	Report which of the following are publicly available and where they can be found: template data collection forms; data extracted from included studies; data used for all analyses; analytic code; any other materials used in the review.	data used for all analyses

References

1. Barlow, J., and T. P. J. Solomon. "Conditioned Media from Contracting Skeletal Muscle Potentiates Insulin Secretion and Enhances Mitochondrial Energy Metabolism of Pancreatic Beta-Cells." *Metabolism* 91 (2019): 1-9.
2. Beiter, T., J. Hudemann, C. Burgstahler, A. M. Niess, and B. Munz. "Effects of Extracellular Orotic Acid on Acute Contraction-Induced Adaptation Patterns in C2c12 Cells." *Mol Cell Biochem* 448, no. 1-2 (2018): 251-63.
3. Guigni, Blas A., Dennis K. Fix, Joseph J. Bivona 3rd, Bradley M. Palmer, James A. Carson, and Michael J. Toth. "Electrical Stimulation Prevents Doxorubicin-Induced Atrophy and Mitochondrial Loss in Cultured Myotubes." *American Journal of Physiology-Cell Physiology* 317, no. 6 (2019): C1213-C28.
4. Broholm, C., M. J. Laye, C. Brandt, R. Vadalasetty, H. Pilegaard, B. K. Pedersen, and C. Scheele. "Lif Is a Contraction-Induced Myokine Stimulating Human Myocyte Proliferation." *J Appl Physiol (1985)* 111, no. 1 (2011): 251-9.
5. Burch, N., A. S. Arnold, F. Item, S. Summermatter, G. Brochmann Santana Santos, M. Christe, U. Boutellier, M. Toigo, and C. Handschin. "Electric Pulse Stimulation of Cultured Murine Muscle Cells Reproduces Gene Expression Changes of Trained Mouse Muscle." *PLoS One* 5, no. 6 (2010): e10970.
6. Christensen, C. S., D. P. Christensen, M. Lundh, M. S. Dahllof, T. N. Haase, J. M. Velasquez, M. J. Laye, T. Mandrup-Poulsen, and T. P. Solomon. "Skeletal Muscle to Pancreatic Beta-Cell Cross-Talk: The Effect of Humoral Mediators Liberated by Muscle Contraction and Acute Exercise on Beta-Cell Apoptosis." *J Clin Endocrinol Metab* 100, no. 10 (2015): E1289-98.
7. Laurens, Claire, Anisha Parmar, Enda Murphy, Deborah Carper, Benjamin Lair, Pauline Maes, Julie Vion, Nathalie Boulet, Coralie Fontaine, Marie Marquès, Dominique Larrouy, Isabelle Harant, Claire Thalamas, Emilie Montastier, Sylvie Caspar-Bauguil, Virginie Bourlier, Geneviève Tavernier, Jean-Louis Grolleau, Anne Bouloumié, Dominique Langin, Nathalie Viguerie, Fabrice Bertile, Stéphane Blanc,

Isabelle de Glisezinski, Donal O’Gorman, and Cedric Moro. "Growth and Differentiation Factor 15 Is Secreted by Skeletal Muscle During Exercise and Promotes Lipolysis in Humans." *JCI Insight* 5, no. 6 (2020).
8. Connor, M. K., I. Irrcher, and D. A. Hood. "Contractile Activity-Induced Transcriptional Activation of Cytochrome C Involves Sp1 and Is Proportional to Mitochondrial Atp Synthesis in C2c12 Muscle Cells." *J Biol Chem* 276, no. 19 (2001): 15898-904.
9. Feng, Y. Z., N. Nikolic, S. S. Bakke, E. T. Kase, K. Guderud, J. Hjelmesaeth, V. Aas, A. C. Rustan, and G. H. Thoresen. "Myotubes from Lean and Severely Obese Subjects with and without Type 2 Diabetes Respond Differently to an in Vitro Model of Exercise." *Am J Physiol Cell Physiol* 308, no. 7 (2015): C548-56.
10. Fernandez-Verdejo, R., A. M. Vanwynsberghe, T. Hai, L. Deldicque, and M. Francaux. "Activating Transcription Factor 3 Regulates Chemokine Expression in Contracting C2c12 Myotubes and in Mouse Skeletal Muscle after Eccentric Exercise." *Biochem Biophys Res Commun* 492, no. 2 (2017): 249-54.
11. Fujita, H., K. Shimizu, and E. Nagamori. "Novel Method for Measuring Active Tension Generation by C2c12 Myotube Using Uv-Crosslinked Collagen Film." *Biotechnol Bioeng* 106, no. 3 (2010): 482-9.
12. Furuichi, Y., Y. Manabe, M. Takagi, M. Aoki, and N. L. Fujii. "Evidence for Acute Contraction-Induced Myokine Secretion by C2c12 Myotubes." *PLoS One* 13, no. 10 (2018): e0206146.
13. Gong, H., L. Liu, C. X. Ni, Y. Zhang, W. J. Su, Y. J. Lian, W. Peng, J. P. Zhang, and C. L. Jiang. "Dexamethasone Rapidly Inhibits Glucose Uptake Via Non-Genomic Mechanisms in Contracting Myotubes." *Arch Biochem Biophys* 603 (2016): 102-9.
14. Horie, M., E. Warabi, S. Komine, S. Oh, and J. Shoda. "Cytoprotective Role of Nrf2 in Electrical Pulse Stimulated C2c12 Myotube." *PLoS One* 10, no. 12 (2015): e0144835.
15. Kubis, H. P., R. J. Scheibe, J. D. Meissner, G. Hornung, and G. Gros. "Fast-to-Slow Transformation and Nuclear Import/Export Kinetics of the Transcription Factor Nfatc1 During Electrostimulation of Rabbit Muscle Cells in Culture." *J Physiol* 541, no. Pt 3 (2002): 835-47.
16. Lambertucci, R. H., R. Silveira Ldos, S. M. Hirabara, R. Curi, G. Sweeney, and T. C. Pithon-Curi. "Effects of Moderate Electrical Stimulation on Reactive Species Production by Primary Rat Skeletal Muscle Cells: Cross Talk between Superoxide and Nitric Oxide Production." *J Cell Physiol* 227, no. 6 (2012): 2511-8.
17. Lee, I. H., Y. J. Lee, H. Seo, Y. S. Kim, J. O. Nam, B. D. Jeon, and T. D. Kwon. "Study of Muscle Contraction Induced by Electrical Pulse Stimulation and Nitric Oxide in C2c12 Myotube Cells." *J Exerc Nutrition Biochem* 22, no. 1 (2018): 22-28.
18. Li, Z., Y. Yue, F. Hu, C. Zhang, X. Ma, N. Li, L. Qiu, M. Fu, L. Chen, Z. Yao, P. J. Bilan, A. Klip, and W. Niu. "Electrical Pulse Stimulation Induces Glut4 Translocation in C2c12 Myotubes That Depends on Rab8a, Rab13, and Rab14." *Am J Physiol Endocrinol Metab* 314, no. 5 (2018): E478-E93.
19. Manabe, Y., S. Miyatake, M. Takagi, M. Nakamura, A. Okeda, T. Nakano, M. F. Hirshman, L. J. Goodyear, and N. L. Fujii. "Characterization of an Acute Muscle Contraction Model Using Cultured C2c12 Myotubes." *PLoS One* 7, no. 12 (2012): e52592.

20. Martin, N. R. W., M. C. Turner, R. Farrington, D. J. Player, and M. P. Lewis. "Leucine Elicits Myotube Hypertrophy and Enhances Maximal Contractile Force in Tissue Engineered Skeletal Muscle in Vitro." *J Cell Physiol* 232, no. 10 (2017): 2788-97.
21. MCARDLE, A. "Contractile Activity-Induced Oxidative Stress: Cellular Origin and Adaptive Responses." *Am J Physiol Cell Physiol* 280 (2001): 621-27.
22. Nieuwoudt, S., A. Mulya, C. E. Fealy, E. Martelli, S. Dasarathy, S. V. Naga Prasad, and J. P. Kirwan. "In Vitro Contraction Protects against Palmitate-Induced Insulin Resistance in C2c12 Myotubes." *Am J Physiol Cell Physiol* 313, no. 5 (2017): C575-C83.
23. Nikolic, N., S. S. Bakke, E. T. Kase, I. Rudberg, I. Flo Halle, A. C. Rustan, G. H. Thoresen, and V. Aas. "Electrical Pulse Stimulation of Cultured Human Skeletal Muscle Cells as an in Vitro Model of Exercise." *PLoS One* 7, no. 3 (2012): e33203.
24. Løvsletten, Nils, Arild Rustan, Claire Laurens, Hege Thoresen, Cedric Moro, and Nataša Nikolić. "Primary Defects in Lipid Handling and Resistance to Exercise in Myotubes from Obese Donors with and without Type 2 Diabetes." *Applied Physiology, Nutrition, and Metabolism* 45 (2019).
25. Park, S., K. D. Turner, D. Zheng, J. J. Brault, K. Zou, A. B. Chaves, T. S. Nielsen, C. J. Tanner, J. T. Treebak, and J. A. Houmard. "Electrical Pulse Stimulation Induces Differential Responses in Insulin Action in Myotubes from Severely Obese Individuals." *J Physiol* 597, no. 2 (2019): 449-66.
26. Pattamaprapanont, P., C. Garde, O. Fabre, and R. Barres. "Muscle Contraction Induces Acute Hydroxymethylation of the Exercise-Responsive Gene Nr4a3." *Front Endocrinol (Lausanne)* 7 (2016): 165.
27. Pattwell, D. M., A. McArdle, J. E. Morgan, T. A. Patridge, and M. J. Jackson. "Release of Reactive Oxygen and Nitrogen Species from Contracting Skeletal Muscle Cells." *Free Radic Biol Med* 37, no. 7 (2004): 1064-72.
28. Raschke, S., K. Eckardt, K. Bjorklund Holven, J. Jensen, and J. Eckel. "Identification and Validation of Novel Contraction-Regulated Myokines Released from Primary Human Skeletal Muscle Cells." *PLoS One* 8, no. 4 (2013): e62008.
29. Raschke, S., M. Elsen, H. Gassenhuber, M. Sommerfeld, U. Schwahn, B. Brockmann, R. Jung, U. Wisloff, A. E. Tjonna, T. Raastad, J. Hallen, F. Norheim, C. A. Drevon, T. Romacho, K. Eckardt, and J. Eckel. "Evidence against a Beneficial Effect of Irisin in Humans." *PLoS One* 8, no. 9 (2013): e73680.
30. Sato, S., M. Nomura, I. Yamana, A. Uchiyama, Y. Furuichi, Y. Manabe, and N. L. Fujii. "A New in Vitro Muscle Contraction Model and Its Application for Analysis of Mtorc1 Signaling in Combination with Contraction and Beta-Hydroxy-Beta-Methylbutyrate Administration." *Biosci Biotechnol Biochem* 83, no. 10 (2019): 1851-57.
31. Scheler, M., M. H. de Angelis, H. Al-Hasani, H. U. Haring, C. Weigert, and S. Lehr. "Methods for Proteomics-Based Analysis of the Human Muscle Secretome Using an in Vitro Exercise Model." *Methods Mol Biol* 1295 (2015): 55-64.
32. Tarum, J., M. Folkesson, P. J. Atherton, and F. Kadi. "Electrical Pulse Stimulation: An in Vitro Exercise Model for the Induction of Human Skeletal Muscle Cell Hypertrophy. A Proof-of-Concept Study." *Exp Physiol* 102, no. 11 (2017): 1405-13.
33. Thelen, Marc H.M. "Electrical Stimulation of C2c12 Myotubes Induces Contractions and Represses Thyroid-Hormone-Dependent Transcription of the Fast-Type Sarcoplasmic-Reticulum Ca2+-Atpase Gene." *Biochemistry Journal* 321 (1997): 845-48.

34. Son, Y. H., S. M. Lee, S. H. Lee, J. H. Yoon, J. S. Kang, Y. R. Yang, and K. S. Kwon. "Comparative Molecular Analysis of Endurance Exercise in Vivo with Electrically Stimulated in Vitro Myotube Contraction." *J Appl Physiol (1985)* 127, no. 6 (2019): 1742-53.
35. Yue, Yingying, Chang Zhang, Xuejiao Zhang, Shitian Zhang, Qian Liu, Fang Hu, Xiaoting Lv, Hanqi Li, Jianming Yang, Xinli Wang, Liming Chen, Zhi Yao, Hongquan Duan, and Wenyan Niu. "An Ampk/Axin1-Rac1 Signaling Pathway Mediates Contraction-Regulated Glucose Uptake in Skeletal Muscle Cells." *American Journal of Physiology-Endocrinology and Metabolism* 318, no. 3 (2020): E330-E42.
36. Valero-Breton, M., G. Warnier, M. Castro-Sepulveda, L. Deldicque, and H. Zbinden-Foncea. "Acute and Chronic Effects of High Frequency Electric Pulse Stimulation on the Akt/Mtor Pathway in Human Primary Myotubes." *Front Bioeng Biotechnol* 8 (2020): 565679.
37. Chaves, A. B., E. R. Miranda, J. T. Mey, B. K. Blackburn, K. N. Z. Fuller, B. Stearns, A. Ludlow, D. L. th Williamson, J. A. Houmard, and J. M. Haus. "Exercise Reduces the Protein Abundance of Txnip and Its Interacting Partner Redd1 in Skeletal Muscle: Potential Role for a Pka-Mediated Mechanism." *J Appl Physiol (1985)* 132, no. 2 (2022): 357-66.
38. Kugler, B. A., W. Deng, B. Francois, M. Anderson, J. M. Hinkley, J. A. Houmard, P. N. Gona, and K. Zou. "Distinct Adaptations of Mitochondrial Dynamics to Electrical Pulse Stimulation in Lean and Severely Obese Primary Myotubes." *Med Sci Sports Exerc* 53, no. 6 (2021): 1151-60.
39. Nakamura, T., S. Takagi, D. Okuzaki, S. Matsui, and T. Fujisato. "Hypoxia Transactivates Cholecystokinin Gene Expression in 3d-Engineered Muscle." *J Biosci Bioeng* 132, no. 1 (2021): 64-70.
40. Small, L., A. Altintas, R. C. Laker, A. Ehrlich, P. Pattamaprapanont, J. Villarroel, N. J. Pillon, J. R. Zierath, and R. Barres. "Contraction Influences Per2 Gene Expression in Skeletal Muscle through a Calcium-Dependent Pathway." *J Physiol* 598, no. 24 (2020): 5739-52.
41. Tamura, Y., K. Kouzaki, T. Kotani, and K. Nakazato. "Electrically Stimulated Contractile Activity-Induced Transcriptomic Responses and Metabolic Remodeling in C2c12 Myotubes: Twitch Vs. Tetanic Contractions." *Am J Physiol Cell Physiol* 319, no. 6 (2020): C1029-C44.
